# The emerging role of furin in neurodegenerative and neuropsychiatric diseases

**DOI:** 10.1186/s40035-022-00313-1

**Published:** 2022-08-23

**Authors:** Yi Zhang, Xiaoqin Gao, Xue Bai, Shanshan Yao, Yan-Zhong Chang, Guofen Gao

**Affiliations:** 1 Ministry of Education Key Laboratory of Molecular and Cellular Biology, Laboratory of Molecular Iron Metabolism, College of Life Sciences, Hebei Normal University, Shijiazhuang, 050024 China; 2 Shijiazhuang People’s Hospital, Hebei Medical University, Shijiazhuang, 050027 China

**Keywords:** Furin, Proteolytic cleavage, Neurodegenerative disease, Neuropsychiatric disease, Brain-derived neurotrophic factor

## Abstract

Furin is an important mammalian proprotein convertase that catalyzes the proteolytic maturation of a variety of prohormones and proproteins in the secretory pathway. In the brain, the substrates of furin include the proproteins of growth factors, receptors and enzymes. Emerging evidence, such as reduced *FURIN* mRNA expression in the brains of Alzheimer’s disease patients or schizophrenia patients, has implicated a crucial role of furin in the pathophysiology of neurodegenerative and neuropsychiatric diseases. Currently, compared to cancer and infectious diseases, the aberrant expression of furin and its pharmaceutical potentials in neurological diseases remain poorly understood. In this article, we provide an overview on the physiological roles of furin and its substrates in the brain, summarize the deregulation of furin expression and its effects in neurodegenerative and neuropsychiatric disorders, and discuss the implications and current approaches that target furin for therapeutic interventions. This review may expedite future studies to clarify the molecular mechanisms of furin deregulation and involvement in the pathogenesis of neurodegenerative and neuropsychiatric diseases, and to develop new diagnosis and treatment strategies for these diseases.

## Introduction

Furin is the first proprotein convertase (PC) found in mammals in 1990 [1]. It catalyzes the proteolytic maturation of large numbers of prohormones and proproteins in the secretory pathway compartments [1–3]. The substrates of furin include hormones, cytokines, growth factors and enzymes, which play important roles in cell proliferation, anti-apoptosis, immunity and inflammation [1]. Furin also participates in the proteolytic processing of proteins in viruses and bacteria [4], such as the maturation of SARS-CoV-2 spike protein [5–7]. Thus, aberrant activity of furin has been found to be associated with a strikingly diverse range of pathological events, including cancer, cardiovascular disorders, infectious diseases and neurological diseases [4, 8–10]. Among these disorders, the role of furin in neurological diseases is the most poorly understood.

In the brain, the proprotein substrates cleaved by furin in vivo include precursors of growth factors such as brain-derived neurotrophic factor (BDNF) and nerve growth factor (NGF) [11, 12], α- and β-secretases [13, 14], multiple matrix metalloproteases (MMPs) [15, 16], and other enzymes and receptors [1, 17, 18]. Since these substrates play vital roles in neuronal survival, axon growth, dendritic development, synaptogenesis, neurodegeneration and inflammation [19–22], a stable activity of furin is crucial for maintaining the homeostasis of the central nervous system.

A growing body of evidence has suggested that alterations of furin expression and abnormal cleavage of its substrates contribute to the pathophysiological mechanisms of neurodegenerative and neuropsychiatric diseases. Reduced expression of *FURIN* mRNA has been found in the brains of Alzheimer’s disease (AD) patients [13], and decreased protein levels of furin are found in the cortex of AD mice [23]. The *FURIN* mRNA expression is decreased in the prefrontal cortex of schizophrenia (SCZ) patients [24], whereas increased protein levels of furin are found in the temporal cortex of epilepsy patients [25]. Moreover, studies have also shown that increasing furin expression in the mouse brain enhances BDNF maturation and promotes dendritic spine density and memory in transgenic mice [26], and that inhibiting furin expression reduces the spontaneous rhythmic electrical activity of cerebral neurons and suppresses epileptic seizure activity in epileptic mice [25]. These findings indicate the involvement of furin dysregulation in these neurological disorders, leading to increased interest in furin as a potential biomarker for diagnosis of or as a therapeutic target for treatment of neurological disorders.

In this review, we present an overview on the physiological roles of furin in the brain and deregulations of furin expression and its substrates in neurodegenerative and neuropsychiatric disorders, such as AD, Parkinson’s disease (PD), epilepsy, cerebral ischemia, SCZ and depression. We further discuss the implications of these findings and current approaches that target furin for therapeutic interventions.

## Overview of furin

### Gene structure and transcription of *FURIN*

**Fig. 1:**
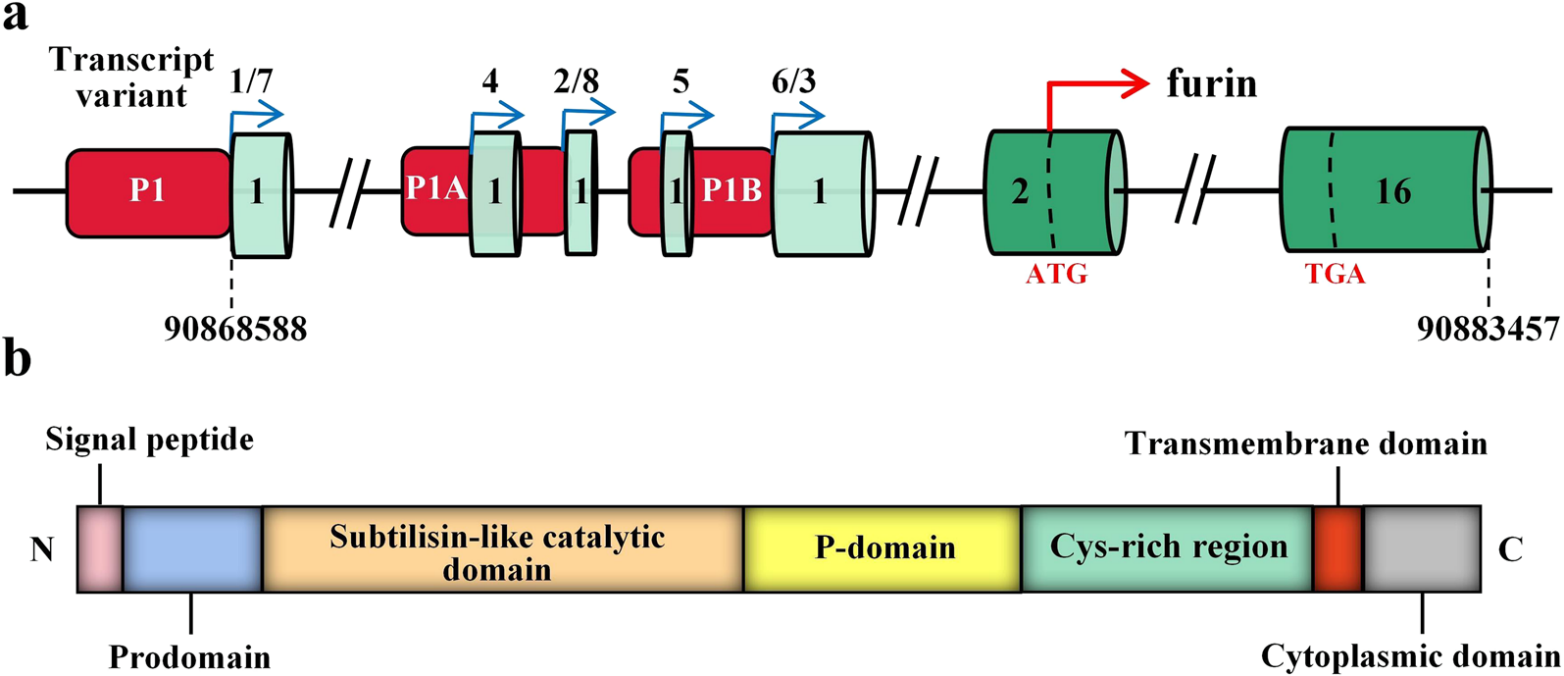
Human *FURIN* gene and furin protein structures. **a** The human *FURIN* gene consists of 16 exons and encodes eight different transcript variants driven by three known promoters, P1, P1A and P1B. Exons are shown as green boxes and introns are shown as lines. The red boxes indicate the three promoter regions. The blue arrows indicate the positions where different transcripts start. The red arrow indicates the translational start, and the start codon (ATG) and stop codon (TGA) are marked with dotted lines. **b** Furin protein contains an N-terminal signal peptide, a prodomain, a subtilisin-like catalytic domain, a middle P-domain, a cysteine-rich region, a transmembrane helix domain and a C-terminal cytoplasmic domain

Furin was identified in 1990 as the first mammalian PC that catalyzes the proteolytic maturation of prohormones and proproteins of neurotrophic factors, receptors and enzymes, serum proteins and pathogen molecules [1–3]. The human *FURIN* gene is located at chromosome 15q26.1, an open reading frame upstream of the fes/fps proto-oncogene [27]. It has attracted more attention after being discovered as the first mammalian homologue of yeast Kex2 [4, 28, 29]. As shown in Fig. 1a, the human *FURIN* gene consists of 16 exons and encodes eight different transcript variants driven by three known promoters, P1, P1A and P1B [30, 31]. The respective transcripts differ only in the first untranslated exon and therefore generate identical furin precursor proteins [30, 32]. While the P1A and P1B promoters resemble those of constitutively expressed housekeeping genes, the P1 promoter is predicted to bind to many different transcription factors, including hypoxia-inducible factor-1 (HIF-1), C/EBPβ, and CREB (cAMP-responsive element binding protein) [33–36].

Several intracellular and extracellular factors have been reported to regulate *FURIN* expression at the transcriptional level. Hypoxia remarkably increases the expression of *FURIN* mRNA via stabilizing HIF-1 and enhancing its binding to hypoxia-responsive element site at the P1 promoter [37]. Iron deficiency also upregulates *FURIN* transcription  through stabilization of HIF-1α [35], whereas iron overload inhibits furin expression in a non-HIF-1α-dependent manner [35]. Transforming growth factor beta1 (TGFβ1) can induce transactivation of the *FURIN* P1 promoter through binding to Sma- and Mad-related protein 2 (SMAD2) and SMAD4 in complex with other DNA-binding partners, creating a constitutive activation/regulation positive feedback loop between TGFβ1 and furin [38]. Furthermore, extracellular regulated protein kinase 1 has been found to mediate the TGFβ–furin feedback loop in glioma-initiating cells [39]. In addition, bone morphogenetic protein 2 increases the transcription and translation of furin in human granulosa lutein cells by the activin receptor-like kinase (ALK)2/ALK3-SMAD4 signaling pathway [40].

### Protein structure and expression of furin

Furin is a type I transmembrane protein and belongs to the subtilisin-like convertase family [1]. It is a calcium-dependent endoserine protease [8]. Furin protein is composed of a signal peptide, a prodomain, a subtilisin-like catalytic domain, a middle P-domain, a cysteine-rich region, a transmembrane helix domain and a cytoplasmic domain (Fig. 1b) [41]. The large extracellular region of furin has an overall homology with the same region of other members of the PC family [1]. The signal peptide directs translocation of the ~ 104-kDa pro-enzyme into the endoplasmic reticulum (ER), where the first cleavage in the inhibitory prodomain takes place via autocatalytic cleavage by the catalytic domain [42–44]. The second cut in the prodomain is made during trafficking of the propeptide-furin complex within the mildly acidic trans-Golgi network/endosomal system, which yields the active ~ 81-kDa mature enzyme. Furin circulates between the trans-Golgi network, cell surface and endosomes, in a tightly regulated manner, to catalyze various proproteins in different cellular components [43, 45, 46].

Furin is ubiquitously expressed in vertebrates and many invertebrates [9, 47, 48]. However, its mRNA and protein levels vary depending on the tissue and cell type [49–53]. *FURIN* has been found at high mRNA levels in the salivary gland, placenta, liver and bone marrow, and high protein levels in the brain, salivary gland, pancreas, kidney and placenta [49–53]. However, almost no expression is detected in skin, muscle and adipose tissues [49, 50], although substrates of furin have been identified in human adipose tissues [54]. In normal single cells, high expression of *FURIN* mRNA is identified in hepatocytes, exocrine glandular cells, pancreatic endocrine cells and syncytiotrophoblasts [50, 53]. This tissue- and cell-specific expression pattern of furin infers the different functions of furin in different tissues and organ systems.

### Function of furin

Furin cleaves proproteins at the consensus site of Arg–X–Lys/Arg–Arg or Arg–X–X–Arg (X refers to any amino acid) [55, 56], and the cut is positioned after the carboxyl-terminal Arg residue [56]. The substrates cleaved by furin include a variety of precursor proteins within the secretory pathway, including hormones, growth factors and their receptors, neuropeptides, enzymes, adhesion molecules, metalloproteinases, bacterial toxins and viral glycoproteins [8, 29, 33]. As these molecules participate in many important cellular events, mouse embryos lacking *Furin* will die between days 10.5 and 11.5, with notable defects in ventral closure and axial rotation [57]. Deregulations of furin expression are found in diverse pathological conditions, including cancer, diabetes, cardiovascular disorders, inflammation and neurological diseases [10, 58–62].

## Furin and its substrates in the brain

### Furin expression in the brain

Brain is one of the organs that show the highest level of furin protein [50], particularly in the cerebral cortex, hippocampus and cerebellum, where the furin level is as high as that in the salivary gland [50]. Moreover, it has been reported that in the brains of epilepsy patients and epileptic mice, furin is predominantly expressed in neurons in the cortex and hippocampus, but barely in glial cells [25]. Double immunofluorescence staining showed a neuron-specific pattern of furin expression in the hippocampal CA3 and dentate gyrus (DG) regions in wild-type mice [63]. The neuron-specific expression may be related to the essential functions of furin in neurons. In addition, it has been reported that furin expression in glial cells may be increased in some pathological conditions as shown by the increase of furin expression in cultured rat astrocytes exposed to oxygen–glucose deprivation [64].

### Substrates cleaved by furin in the brain

**Fig. 2:**
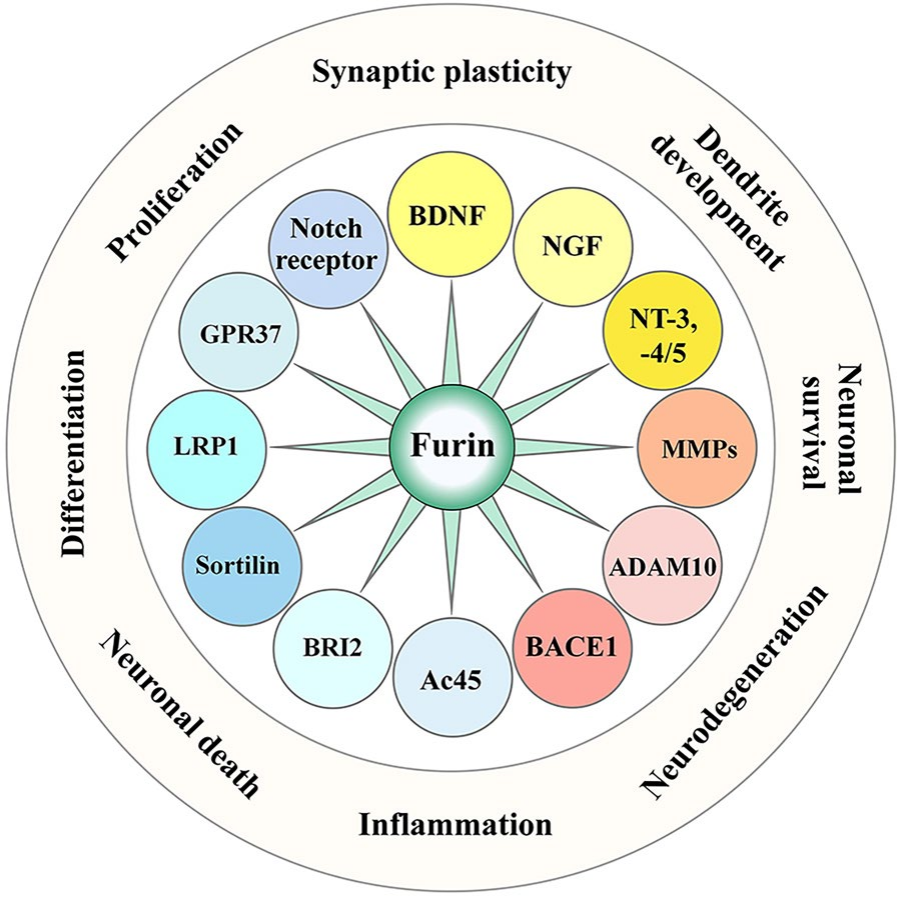
Activities mediated by furin and its substrates in the brain. The substrates of furin include growth factors such as BDNF and NGF, proteases such as MMPs, ADAMs and BACE1, and receptors such as Notch, LRP1, GPR37, sortilin, BRI2 and Ac45. They participate in diverse biological processes in the brain, including neuronal survival and death, proliferation and differentiation, dendritic development, synaptic plasticity, inflammation and neurodegeneration

In the brain, the substrates proteolytically cleaved by furin include growth factors such as BDNF and NGF, proteases such as multiple MMPs, a disintegrin and metalloproteases (ADAMs) and beta-site APP cleaving enzyme 1 (BACE1), and receptors such as Notch receptor, low-density lipoprotein receptor-related protein 1 (LRP1), G protein-coupled receptor (GPR37) sortilin, integral membrane protein 2B (BRI2) and Ac45. Furin and its substrates potentially play important roles in diverse biological processes in the brain, including neuronal survival, differentiation, axonal outgrowth, dendritic development, synaptogenesis, inflammation and neurodegeneration (Fig. 2).

#### BDNF

BDNF is a member of the neurotrophin family, which is widely distributed and extensively expressed in the brain [65–67]. BDNF is synthesized as pre-proBDNF and folded in the ER [68]. The pre-proBDNF harbors a signal peptide, a pro-domain and a mature domain [69], and is transported to the Golgi apparatus where it is converted into a full-length proBDNF (~ 32 kDa) after removal of the signal peptide [59]. The proBDNF is then cleaved by the protease furin to release the pro-domain and generate the biologically active ~ 14-kDa mature BDNF (mBDNF) [70]. The proBDNF can also be secreted into the extracellular space and then catalyzed by the extracellular proteases, such as MMPs [71, 72]. Furin is found to have higher efficiency than other PCs in cleavage of proBDNF in cultured rat astrocytes, and its aberrant activity leads to a significant change in mBDNF expression [64]. In terms of function, mBDNF binds to the tropomyosin-related receptor kinase B (TrkB) [73] and triggers downstream intracellular signaling pathways, including the phosphatidylinositol 3-kinase/protein kinase B (PI3K/Akt), the phospholipase C-γ/calcium-dependent protein kinase (PLCγ/CaMK), and the mitogen-activated protein kinase (MAPK)/ERK pathways [22, 74–76]. These signaling pathways mediate transcription of genes essential for neuronal survival, differentiation, axonal outgrowth, dendritic spine development, hippocampal long-term potentiation (LTP) and synaptic plasticity [22, 49, 74–76]. In contrast, proBDNF binds to p75 neurotrophic receptor (p75NTR) and induces apoptosis, spine shrinkage and long-term depression facilitation [77, 78]. Therefore, imbalances between proBDNF and mBDNF are involved in pathophysiological mechanisms of neurodegenerative diseases, as well as neuropsychiatric diseases [22, 73, 76, 79–81].

#### NGF

NGF is the first identified member of the neurotrophin family [82]. Like other proneurotrophins, the ~ 30-kDa proNGF is synthesized in the ER [83]. Its pro-domain is cleaved mainly intracellularly in the trans-Golgi network by furin, rather than in secretory granules by other PCs [84, 85], releasing the mature NGF (mNGF, ~ 17 kDa) [86, 87]. Similar to BDNF, proNGF and mNGF also differ significantly in receptor interaction properties and bioactivity. The mNGF binds to tropomyosin-related receptor kinase A (TrkA) and promotes cell survival, differentiation, growth and maintenance of specific types of neurons [88–90], whereas the proNGF binds to p75NTR with a high affinity and mediates neuronal cell death [91–93]. The balance between proNGF and mNGF levels is a key determinant of homeostasis in the brain, and disruption of the balance is associated with diseases such as epilepsy, AD, and ischemic stroke [94–96].

#### Other neurotrophins

The third type of growth factors of the neurotrophin family includes neurotrophin-3 (NT-3) and neurotrophin-4/5 (NT-4/5) [97, 98]. They are also synthesized as ~ 31–35-kDa precursors, and in turn proteolytically cleaved to release biologically active mature neurotrophins (~ 13–21 kDa) [84]. Similarly, intracellular cleavage of proneurotrophins is accomplished by furin [99]. The mature neurotrophins then bind to their corresponding receptors, the Trk family of receptor tyrosine kinases, and regulate neuronal survival and synaptic plasticity [100, 101]. Aberrant expressions of NT-3 and NT-4/5 participate in pathophysiological conditions including motor dysfunction, cognitive decline, stroke, and SCZ [102–107].

#### MMPs

MMPs are a family of zinc-dependent metalloproteases [108], with many members being reported to be expressed in the brain, such as MMP-1, MMP-2, MMP-3, MMP-7, MMP-9, MMP-14, and MMP-24 [108]. MMP-1 is expressed in both glia and neurons in the cortex, hippocampus and cerebellum [108, 109]; MMP-2 is mainly expressed in astrocytes [110]; MMP-3 is expressed in glia and neurons in the cerebellum, striatum and hippocampus [111]; and MMP-9 is mainly expressed in neurons in the cerebral cortex, hippocampus and cerebellum [112, 113]. Typically, MMPs consist of a signal peptide, a propeptide sequence, a catalytic metalloproteinase domain with zinc, a hinge region, and a hemopexin domain [114]. The signal peptide is removed in ER [115], and the propeptide is cut off by furin or other PCs at a furin-like recognition motif [116–118]. The MMPs are thus activated inside the cell before secretion or exposure to cell surface [119]. MMP-1 is shown to enhance the proliferation and neuronal differentiation of adult hippocampal neural progenitor cells via activating protease activated receptor 1 and subsequently increasing the cytoplasmic Ca^2+^ concentration [120, 121]. MMP-2 regulates astrocyte motility in connection with the actin cytoskeleton and integrins [122]. MMP-3 has a very broad range of substrates in the brain [123], and is upregulated in many pathological conditions, inducing neuroinflammation and apoptosis [124]. MMP-9 is specifically shown to regulate synaptic plasticity in the hippocampus by gain- and loss-of-function studies in vitro [125, 126]. Altered concentrations of MMP-3 and MMP-9 have been found in AD patients, indicating their involvement in AD pathophysiology [127]. MMP-1, MMP-2, MMP-9 and MMP-14 can cleave recombinant α-synuclein [128, 129]. Elevated levels of MMP-2 and MMP-3 have been identified in dopaminergic (DA) neurons in the substantia nigra in PD patients and animal models [129–131].

#### ADAM10

ADAMs are another major family of zinc-dependent metalloproteases involved in limited proteolysis and shedding [132]. In the brain, ADAM10 is mainly expressed in neurons [133], and is involved in the proteolytic processing of a variety of cell surface receptors and signaling molecules [134]. ADAM10 is synthesized in the ER as an inactive zymogen with a structure comprising a prodomain, a zinc-binding metalloprotease domain, a disintegrin domain, a cysteine-rich domain, a transmembrane domain and a C-terminal domain [133]. Furin cleaves the ~ 90 kDa pro-ADAM10, yielding a full-length active ADAM10 (∼65 kDa) [135], and after C-terminal shedding, a soluble ∼55-kDa ADAM10 is released [136]. ADAM10 has α-secretase activity [137]. It cleaves amyloid-β precursor protein (APP) to generate the soluble αAPP fragment (sAPPα) rather than the neurotoxic amyloid-β (Aβ), playing a protective role in AD [138].

#### BACE1

BACE1 is the major β-secretase that cleaves APP to generate Aβ [139]. BACE1 is a transmembrane aspartic protease, structurally similar to the pepsin family [140], containing two active catalytic site motifs in the luminal domain [141]. Like other aspartic proteases, BACE1 is synthesized as a precursor protein containing a N-terminal propeptide domain that is removed during maturation of the enzyme [142]. Furin or a furin-like PC is responsible for cleaving the BACE1 proprotein to yield the mature enzyme with the highest β-secretase activity [143]. Like APP, BACE1 is highly expressed in the brain [144]. Significant increases of BACE1 enzymatic activity and protein concentration have been detected in brain tissues, cerebrospinal fluid (CSF) and serum of AD patients and subjects with mild cognitive impairment [145–147]. BACE1 inhibitors have demonstrated therapeutic effects in preventing the initial cleaving events of APP in AD animal models [148–153].

#### Notch receptor

The Notch gene family encodes transmembrane receptors of ~ 300 kDa that are involved in cell-fate determination in vertebrates and invertebrates [154, 155]. The proteolytic processing of Notch receptor precursor is an essential step in the formation of biologically active Notch receptors. The constitutive processing of murine Notch1 requires a furin-like convertase, and mutations in the furin-cleavage site completely abolishes the proteolysis of the Notch1 receptor [155]. In the developing brain, activation of Notch receptors upon ligand binding is involved in the preservation of neural progenitors and inhibition of neurogenesis [156, 157]. In the adult brain, Notch signaling influences neuronal apoptosis, microglial activation and synaptic plasticity [158–161]. Deregulations of Notch signaling are involved in AD, depression, epilepsy, and stroke [159–163].

#### LRP1

LRP1 is a multifunctional receptor that belongs to the low-density lipoprotein receptor family [164]. It is synthesized as a ~ 600-kDa precursor, which is cleaved by furin in the trans-Golgi network and transported to the cell surface as a mature form consisting of α-chain and β-chain [8]. The mature LRP1 is further processed by other enzymes, such as MMPs and γ-secretase, to release the intracellular domain (ICD) [8]. LRP1 is highly expressed in neurons and glia of the brain, and functions to regulate proteinase activity, cytokine activity and cholesterol metabolism [165, 166]. The ligands for LRP1 include Aβ, ApoE and activated α2-macroglobulin [167]. In addition to controlling ligand metabolism, LRP1 can also regulate signaling pathways by coupling with other cell surface receptors or proteins, such as the *N*-methyl-*D*-aspartate (NMDA) receptors [168, 169]. The ICD of LRP1 can be transported into the nucleus, where it contributes to transcriptional regulation of target genes, including interferon-γ [170]. Accumulating evidence from preclinical and animal studies indicates that LRP1 is involved in AD pathogenesis not only by regulating the metabolisms of Aβ and ApoE, but also by influencing synaptic plasticity and inflammation through Aβ-independent pathways [171]. LRP1 is detected at an abundant level in post-synaptic sites of neurons, and it interacts with several synaptic proteins, including postsynaptic density protein 95, NMDA receptor and GluA1 [169, 171–173]. Deletion of LRP1 in neurons has been shown to affect lipid metabolism, leptin signaling, glucose metabolism, insulin signaling and anti-apoptotic signaling, resulting in neuroinflammation, motor dysfunction, and cognitive decline in mice [171, 172, 174, 175]. In addition, LRP1 is also found to modulate stem cell proliferation and survival, astroglial differentiation [176, 177], and oligodendrocyte progenitor cell differentiation [178].

#### GPR37

GPR37 is an orphan G-protein-coupled receptor that is widespread in several brain regions, including cerebral cortex, hippocampus, hypothalamus, midbrain and cerebellum [51]. It has a long extracellular N-terminal ectodomain which is recently demonstrated to be processed by both ADAM10 and furin [179]. The unfolded form of GPR37 is a substrate of parkin, and its intracellular retention leads to ER stress and DA neuronal death, linking to PD [180–182]. GPR37 is also involved in the DA signaling pathway by interacting with the dopamine transporter in mouse striatal presynaptic membranes, thereby modulating dopamine uptake [183]. In addition, GPR37 interacts with adenosine A2A receptors in the hippocampus, localized at the extrasynaptic plasma membrane of dendritic spines, dendritic shafts and axon terminals, regulating adenosinergic signaling [184]. GPR37 is also found in astrocytes and oligodendrocytes, and is demonstrated as a negative regulator of oligodendrocyte differentiation and myelination [185, 186]. Overexpression of GPR37 leads to profound neurodegeneration in animal models, selectively for DA neurons [187], while GPR37-knockout mice also show decreased dopamine levels in the striatum and specific motor deficits [188, 189]. GPR37 knockout also triggers non-motor behavioral phenotypes, such as anxiety and depression-like behaviors, in an age- and gender-dependent manner [190, 191].

#### Sortilin

Sortilin is a type I transmembrane protein that functions as an endocytosis receptor and plays a role in protein sorting and cell signaling [192]. Sortilin is synthesized as an inactive precursor protein, which is cleaved by furin to remove the N-terminal propeptide [193]. The resulting mature protein can be further processed by other proteases to shed its extracellular domain from the cell surface [193]. Sortilin is generally trafficked via the trans-Golgi network, endosomes and plasma membrane, binding to different proteins and directing them to the secretory pathway or for degradation [193]. Sortilin has been reported to function as a neuronal receptor for APP and its cleavage products sAPPα and Aβ [194, 195]. The ICD of sortilin interacts with APP and regulates its lysosomal and lipid raft trafficking [194]. Sortilin also binds to oligomerized Aβ, inducing endocytosis of Aβ and triggering apoptosis [195]. In addition, sortilin is found to be an essential component for transmitting pro-neurotrophin-dependent death signals from p75NTR, thereby playing roles in neuronal apoptosis, aging and brain injury [93, 196, 197]. On the other hand, sortilin has also been found to associate with TrkB receptors, which promotes cell survival [198]. Therefore, sortilin acts as a molecular switch from apoptotic response by interacting with p75NTR to neurotrophic effects via binding to TrkB receptors in neurons. Aberrant activity of sortilin has been found to be associated with the pathogenesis of AD and depression [193, 199, 200].

#### BRI2

BRI2 is a type II transmembrane protein of 266 amino acids, containing an extracellular region, a transmembrane region and a cytoplasmic region [201, 202]. During maturation, the ~ 4-kDa C-terminal propeptide of BRI2 is cleaved by furin at the trans-Golgi compartment, generating the membrane-bound form of mature BRI2 (mBRI2) [203, 204]. The mBRI2 contains an evolutionarily conserved BRICHOS domain that is found to act as a chaperone, facilitating proper folding of BRI2 and preventing Aβ formation [205, 206]. In the human brain, BRI2 is intensively expressed in cortical and hippocampal pyramidal neurons [207]. The BRICHOS domain of BRI2 interacts with APP and inhibits its processing, delaying fibrillation of Aβ [206–209]. Mutations in BRI2 and aberrant BRI2 expression have been reported to be associated with familial British dementia and involved in AD pathogenesis [210–212].

#### Ac45

Ac45, an accessory subunit of the vacuolar-type ATPase (V-ATPase) proton pump, is a type I transmembrane protein that is encoded by *ATP6AP1* in humans [213–215]. Furin catalyzes the processing of Ac45 precursor protein to generate mature Ac45 [216, 217]. *Furin*-knockout β-cells show impaired cleavage of Ac45 [217]. Ac45 is ubiquitously expressed with the highest levels in neuronal and neuroendocrine cells and osteoclasts [218–220], and may be required for proper synaptic vesicle acidification and neurotransmitter release [221]. Ac45-deficient patients not only have immunodeficiency, but also display a spectrum of neurocognitive abnormalities [222]. These indicate that dysfunction of Ac45 can be potentially involved in neurological disorders such as AD and epilepsy.

## Furin in neurodegenerative and neuropsychiatric diseases

So far, many studies have demonstrated associations of deregulation of furin expression with the pathophysiology of several neurodegenerative and neuropsychiatric diseases, as well as with alterations of substrates of furin in these diseases (Table 1).

**Table 1:** Changes in the expression of furin and its substrates in neurodegenerative and neuropsychiatric diseases and the implications

Disease	Patients/animal models	Furin expression	Expression of proteins processed by furin	Implications	References
AD	AD patients	*FURIN* mRNA (brain) ↓		Furin reduction may be closely related to the mechanisms that lead to Aβ production in AD	[13]
AD	Tg2576 mice	*Furin* mRNA (cortex) ↓		Furin reduction downregulates α-secretase activity of ADAM10 and TACE, thereby enhancing Aβ production	[13]
AD	APP-C105 mice	Furin (cortex) ↓	ADAM10 (cortex) ↓	Excess iron induces disruption of furin activity, which in turn reduces α-secretase-dependent APP processing	[23]
AD	AD patients	Furin (plasma) ↓		Increased plasma iron concentration in AD downregulates furin level, impairing the ability of α-secretases to produce sAPPα, resulting in increased Aβ	[237]
AD	AD patients		*BDNF* mRNA (hippocampus) ↓; BDNF (hippocampus) ↓	Deficiency of BDNF may contribute to the progressive atrophy of neurons in AD	[240] [241]
AD	AD patients		*BDNF* mRNA (cortex) ↓; mBDNF (cortex) ↓; mBDNF/proBDNF (cortex) ↓	Imbalanced proBDNF and mBDNF play a role in synaptic loss and cellular dysfunction, leading to cognitive impairment in AD	[241] [242] [243] [244] [245] [246]
AD	Tg2576 mice		mBDNF (hippocampus) ↓; mBDNF/proBDNF (hippocampus) ↓	Abnormal cleavage of BDNF may be involved in AD-related traits triggered by excessive Aβ pathology	[247]
AD	5 × FAD mice		BDNF (hippocampus) ↓	BDNF expression is reduced in 5 × FAD mice at the age of 3 and 7 months, contributing to the impairment of synaptic plasticity and memory	[248] [249]
AD	AD patients		proNGF (cortex) ↑	Decreased processing of proNGF to mNGF may be associated with AD pathology	[94] [250]
AD	AD patients		proNGF (hippocampus) ↑	Alterations in the hippocampal NGF signaling pathway in AD favor proNGF-mediated proapoptotic pathways	[251]
AD	AD patients		Notch1 (hippocampus) ↑	Notch1 is increased in AD and Pick’s disease, where abnormal tau aggregates are present, indicating a possible relationship between tau aggregation and Notch1 expression	[252]
AD	AD patients		MMP-1 (cortex) ↑	Enhanced MMP-1 activity in AD may contribute to the BBB dysfunction seen in AD	[255]
AD	AD patients		*BACE1* mRNA (cortex) ↑ BACE1 (cortex) ↑	Increased BACE1 activity is correlated with Aβ level in AD	[253, 254]
AD	AD patients		BACE1 (CSF) ↑	Increased BACE1 in CSF is a predictor of mild cognitive impairment	[146]
AD	5× FAD mice		MMP-2 (hippocampus) ↑; MMP-9 (hippocampus) ↑; MMP-14 (hippocampus) ↑	Different MMPs involved in APP/Aβ metabolism are differentially regulated in a spatio-temporal manner in the 5× FAD murine model of AD	[256]
AD	AD patients		Sortilin (cortex) ↑	Sortilin functions as a modulator of BACE1 retrograde trafficking and promotes the generation of Aβ	[199]
AD	AD patients		Sortilin (hippocampus) ↑; ProBDNF (hippocampus) ↑; ProBDNF/BDNF (CSF) ↑	ProBDNF-p75/sortilin signaling is an important contributor to the pathogenesis of AD, causing an increase of cell death and impairment of neuronal differentiation	[257]
AD	AD patients		*LRP1* mRNA (cortex) ↑; LRP1 (brain) ↑	LRP1 expression may be upregulated in glial cells due to the neuroinflammation in AD	[258] [259]
AD	AD patients		LRP1 (cortex) ↓	LRP1 pathway may modulate Aβ deposition and AD susceptibility by regulating the removal of soluble Aβ	[260]
AD	APP23 mice		LRP1 (cortex) ↑; LRP1 (cortical blood vessels) ↓	LRP1 increase in the cortex and decrease in vascular endothelial cells may account for an imbalance of Aβ efflux and influx across the BBB in AD mice	[261]
AD	AD patients		BRI2-BRICHOS (hippocampus) ↑; BRI2-APP (hippocampus) ↓	Aberrant processing of BRI2 may promote its deposition and affect its function in halting Aβ aggregation	[212]
PD	LRRK2-overexpressing *Drosophila*	Furin 1 (DA neurons) ↑		LRRK2 enhances furin 1 translation in DA neurons, mediating neurotoxicity in the fly model of PD	[265]
PD	Paraquat-treated *Drosophila*	Furin 1 (DA neurons) ↑		Furin 1 may initiate a cellular program that is central to the process of neurodegeneration	[265]
PD	PD patients		BDNF (CSF) ↑	Altered BDNF level could be involved in the pathophysiology of PD	[267]
PD	PD patients		BDNF (serum) ↓	Lower serum levels of BDNF at early stage may be associated with the pathogenesis of PD	[268] [269]
PD	PD patients		MMP-2 (substantia nigra) ↓	Region-specific alterations of MMPs may contribute to the pathogenesis of PD	[131]
PD	6-Hydroxydopamine-treated rats		MMP-3 (substantia nigra) ↑	Activation of MMP-3 processes the secreted α-synuclein in PD	[129]
PD	PD patients		MMP-1 (serum) ↓	Significantly lower levels of serum MMP-1 were found in PD patients, particularly in females	[270]
PD	PD patients		GPR37 (Lewy bodies in midbrain) ↑	GPR37 may be involved in the formation of Lewy bodies, mediating neurotoxicity in PD	[181]
PD	PD patients		*GPR37* mRNA (substantia nigra) ↑; Ecto-GPR37 (CSF) ↑	Ecto-GPR37 in CSF is a potential biomarker for PD	[182]
Epilepsy	TLE patients	Furin (temporal cortex) ↑		There might be a correlation between furin expression and epilepsy	[25]
Epilepsy	KA-induced epileptic mice; PTZ-kindled epileptic mice	Furin (cortex, hippocampus) ↑		Furin may play a role in regulation of inhibitory synaptic transmission in epileptic mice	[25]
Epilepsy	KA-induced epileptic mice	*Furin* mRNA (hippocampus) ↑	*Ngf* mRNA (hippocampus) ↑; *Bdnf* mRNA (hippocampus) ↑	*Furin* mRNA upregulation appears to be parallel to that of NGF and BDNF mRNAs following KA treatment	[12]
Epilepsy	TLE patients		*BDNF*/*NGF*/*NT-3* mRNA (hippocampus) ↑	There may be associations between increased neurotrophin mRNA levels in granule cells and damage to hippocampal neurons and synaptic plasticity in epilepsy	[276]
Epilepsy	TLE patients		BDNF (temporal cortex) ↑	The activity-dependent expression of BDNF in human subjects potentially contributes to the pathophysiology of human epilepsy	[277]
Epilepsy	Pilocarpine-induced status epileptic mice		ProBDNF (hippocampus) ↑	Rapid increases of proBDNF following epilepsy are due in part to reduced cleavage	[278]
Epilepsy	Rats with limbic seizures induced by electrolytic lesion in DG		*Ngf* mRNA (hippocampus) ↑; *Ngf* mRNA (cortex) ↑	The expression of NGF is affected by unusual physiological activity	[279]
Epilepsy	KA-induced epileptic rats		*Ngf* mRNA (forebrain) ↑	Seizure activity stimulates a transient increase of NGF expression by selective populations of forebrain neurons	[280]
Epilepsy	Pilocarpine-induced status epileptic rats		ProNGF (hippocampus) ↑	High levels of mRNA for both p75 receptors and proNGF are found in the epileptic model rats	[281]
Epilepsy	TLE patients; KA-induced epileptic mice		Notch (hippocampus) ↑	The effect of Notch signaling on seizures can be in part attributed to its regulation of excitatory synaptic activity in CA1 pyramidal neurons	[163]
Epilepsy	Epilepsy patients		MMP-2 (serum) ↓; MMP-3 (serum) ↓	Serum MMP-2 and MMP-3 are potential biomarkers for epilepsy	[283]
Epilepsy	TLE patients;		*MMP2* mRNA (hippocampus) ↑; *MMP3* mRNA (hippocampus) ↑; *MMP14* mRNA (hippocampus) ↑	Increased MMP expression is a prominent hallmark of the human epileptogenic brain	[285]
Epilepsy	Intractable epilepsy patients		MMP-9 (cortex) ↑	Increased MMP-9 immunoreactivity was prominently upregulated at synapses in the cortex of intractable epilepsy patients	[286]
Epilepsy	PTZ-induced kindled epileptic mice		MMP-9 (hippocampus) ↑	MMP-9 is involved in the progression of epilepsy through cleavage of proBDNF to mBDNF in the hippocampus	[287]
Cerebral ischemia	Global ischemic rats	*Furin* mRNA (hippocampus) ↑		Furin may protect hippocampal neurons from ischemic damage	[296]
Cerebral ischemia	Rats after MCAO	*Furin* mRNA (ischemic hemisphere) ↑	*Mmp2* mRNA (ischemic hemisphere) ↑; *Mmp14* mRNA (ischemic hemisphere) ↑	Furin activates MMP-14 and in turn enhances MMP-2 activation, contributing to the disruption of BBB in ischemia	[297]
Cerebral ischemia	Hypoxic-ischemic rats	*Furin* mRNA (ipsilateral cortex) ↓; *Furin* mRNA (ipsilateral hippocampus) ↑ ↓ ↑	BDNF (ipsilateral cortex, hippocampus) ↓; *Mmp9* mRNA (ipsilateral cortex) ↓	BDNF and its related enzymes such as furin play important roles in the pathogenesis of and recovery from hypoxic-ischemic brain damage	[298]
Cerebral ischemia	Rats after MCAO		MMP-2 (ipsilateral cortex, striatum) ↑; MMP-9 (ipsilateral cortex, striatum) ↑	A specific spatial–temporal pattern of expression and activation of MMP-9 and MMP-2 may contribute to extracellular matrix degradation and BBB breakdown after transient focal cerebral ischemia	[299]
Cerebral ischemia	Baboons after MCAO		MMP-2 (basal ganglia) ↑	It is plausible that locally active MMP-2 contributes to early matrix degradation, loss of vascular integrity, neuron injury, and maturation of the ischemic lesion	[300]
Cerebral ischemia	Mice after MCAO		MMP-9 (ischemic regions) ↑	MMP-9 may play an active role in early vasogenic edema development after stroke	[302]
Cerebral ischemia	Rats after MCAO		LRP1-ICD (ischemic areas) ↑	Furin-mediated cleavage of LRP1 and changes in LRP1-ICD localization are involved in ischemic brain injury	[303]
SCZ	SCZ patients	*FURIN* mRNA (prefrontal cortex) ↓		Aberrant gene expression elucidates the functional impact of polygenic risk for SCZ	[304]
SCZ	SCZ patients		*BDNF* mRNA (cortex) ↓; BDNF (cortex) ↓	Cortical neurons may receive less trophic support in SCZ	[312]
SCZ	SCZ patients		*BDNF* mRNA (cortex) ↓	Decreased BDNF/TrkB signaling appears to underlie the dysfunction of inhibitory neurons in SCZ	[313]
SCZ	SCZ patients		BDNF (hippocampus) ↓; NT-3 (cortex) ↓	Alterations in expression of neurotrophic factors could be responsible for neural maldevelopment and disturbed neural plasticity in SCZ	[314]
SCZ	SCZ patients		BDNF (serum) ↓	BDNF may be involved in the pathophysiology of and cognitive impairment in SCZ	[316] [317]
SCZ	Rats with ibotenic acid lesions in the hippocampus		*Bdnf* mRNA (cortex) ↓; *Bdnf* mRNA (hippocampus) ↓	Alterations in BDNF render animals more susceptible to neurodegenerative insults	[318]
SCZ	Dysbindin-1 mutant mice		BDNF (cortex) ↓; BDNF (hippocampus) ↓	BDNF reduction leads to inhibitory synaptic deficits	[319]
SCZ	SCZ patients		NGF (serum) ↓; NT-3 (serum) ↓	SCZ is accompanied by an abnormal neurotrophin profile	[320] [321] [322]
SCZ	SCZ patients		MMP-9 (serum) ↑	Alterations in plasma MMP-9 are a biomarker for SCZ	[323]
SCZ	SCZ patients		MMP-2 (CSF) ↑	Increased CSF MMP-2 levels in SCZ may be associated with brain inflammation	[326]
Depression	MDD patients		BDNF (serum) ↓	Low BDNF levels may play a pivotal role in the pathophysiology of MDD	[327] [328]
Depression	MDD patients		BDNF (serum) ↓; mBDNF/proBDNF (serum) ↓	The changes in serum BDNF, TrkB, proBDNF and p75NTR may provide a diagnostic biomarker for MDD	[329]
Depression	MDD patients		MMP-9 (serum) ↑; MMP-2 (serum) ↓	MMP-2 and MMP-9 are involved in the pathophysiology of major depression	[323]
Depression	Mood disorder patients		MMP-2 (serum) ↓	A change in inflammatory homeostasis, as indicated by MMP-2 and MMP-9, could be related to mood disorders	[330]
Depression	MDD patients		MMP-2 (CSF) ↑; MMP-7 (CSF) ↑; MMP-10 (CSF) ↑	Increased MMP-2 levels in CSF are positively correlated with clinical symptomatic scores in MMD	[326]
Depression	Rats after chronic unpredictable mild stress		*Lrp1* mRNA (hippocampus) ↑; LRP1 (hippocampus) ↑	LRP1 might impair the microtubule dynamics in depressive-like rats and is involved in the development of depression	[331]

MCAO: middle cerebral artery occlusion

### Furin in AD

#### AD overview

AD is a progressive neurodegenerative disease and the main cause of dementia in the elderly, affecting around 6% of the population over the age of 65 [223]. Currently, there is no effective prevention or treatment strategy for AD [20, 224, 225]. The major pathological hallmarks of AD are the accumulation of two aggregated proteins in the brain, Aβ and tau, leading to the formation of extracellular senile plaques and intracellular neurofibrillary tangles (NFTs), respectively [226, 227]. Aβ is produced from proteolytic cleavage of APP by β- and γ-secretases [139]. In contrast, APP cleaved by α-secretase produces sAPPα which shows neurotrophic and neuroprotective functions [226]. Both Aβ senile plaques and NFTs induce neuroinflammation and neuronal apoptosis, contributing to AD pathogenesis [226, 227]. Following Aβ and tau pathology, AD patients further exhibit synaptic damage and neuronal loss, particularly in the cortex and hippocampus, and show cognitive impairments as the disease progresses [228]. In addition to the Aβ cascade hypothesis, many other hypotheses have also been proposed to explain the pathologic process of AD, including the tau hypothesis [229], the blood–brain barrier (BBB) dysfunction hypothesis [230], the metal ion dysregulation hypothesis [231, 232], the inflammation hypothesis [233], the oxidative stress and mitochondrial cascade hypothesis [234, 235], and the insulin resistance hypothesis [236]. However, these hypotheses only explain certain aspects of the disease, and the mechanism of AD pathogenesis remains elusive.

#### Aberrant furin expression in AD

*FURIN* mRNA expression has been detected at a significantly lower level in the brains of AD patients and Tg2576 AD mouse model than in controls [13]. Notably, decreased mRNA expression of *Furin* is observed in cortices of both 4- and 24-month-old Tg2576 mice compared with their littermates, suggesting that furin reduction occurs in a relatively early age (prior to Aβ plaque formation) and may be involved in the pathogenesis of AD [13]. Moreover, this study also showed that injection of *Furin*-adenovirus into Tg2576 mouse brains markedly reduced Aβ production in the infected brain regions, which may be attributed to the enhancement of the α-secretase activity by furin cleavage of ADAM10 and tumor necrosis factor-α converting enzyme (TACE) [13]. Another study also showed decreased expression of furin and ADAM10 in the cortex of APP-C105 mouse model of AD compared to that of non-transgenic controls [23]. Moreover, treadmill exercise could elevate furin expression and suppress Aβ production in the APP-C105 mice [23]. While excess iron in AD brain induces disruption of furin activity, treadmill exercise alleviates cognitive decline and Aβ-induced neuronal cell death by promoting α-secretase-dependent processing of APP through low iron-induced enhancement of furin activity [23].

In addition to furin expression in the brain, the plasma furin also decreases significantly while serum Aβ increases in AD patients [237]. The decrease of plasma furin strongly correlates with the increase of plasma iron, thereby iron overload in plasma was proposed to be a possible contributor to the low level of furin, and the downstream reduction of α-secretase activity might account for the increase of Aβ [237]. Besides, studies have also reported that the bilateral injection of Aβ into the intracerebral ventricle of mice can induce furin expression compensatorily, which subsequently increases NGF via modulation of its maturation [238, 239].

#### Expression of substrates of furin in AD

Many proteins that are proteolytically processed by furin also show altered expression in AD. Numerous studies have indicated that the relative levels of *BDNF* mRNA and proteins are decreased in the hippocampus and cortex in AD patients [240–246]. Particularly, decreased mBDNF/proBDNF ratio has been found in the parietal cortex of subjects with mild cognitive impairment [246], suggesting that reduction of mBDNF occurs in early stages of AD and contributes to the impairment of synaptic plasticity and memory. In addition to AD patients, transgenic AD mouse models also show reduced mBDNF expression and decreased mBDNF/proBDNF levels in the hippocampus [247–249], indicating the involvement of altered cleavage of BDNF in AD pathology.

Similar to BDNF, NGF, Notch1, ADAM10, BACE1, MMPs, LRP1, BRI2 and sortilin also show altered expression or activity in AD. ProNGF increases markedly in the cortex and hippocampus of AD brains [94, 250, 251]. Notably, the increase of proNGF also appears in subjects with mild cognitive impairment [250]. These findings reflect that the decreased processing of proNGF to mNGF is involved in AD pathogenesis. Notch1 expression is increased in the hippocampus of AD patients, which may be linked to tau aggregation [252]. BACE1 expression has been found to be elevated in the cortex and CSF of AD patients as compared to the age-matched normal subjects [146], which is correlated with increased Aβ [253, 254]. MMP-1 levels are significantly elevated in AD patients in all cortical areas, which may contribute to the BBB dysfunction seen in AD [255]. MMP-2, MMP-9 and MMP-14 expression is up-regulated age-dependently in astrocytes and amyloid plaques in the hippocampus of  5× FAD mice [256]. Sortilin protein and the cytoplasmic domain of sortilin are found to be significantly increased in brains of AD patients, which contribute to the pathogenesis of AD by increasing cell death and impairing neuronal differentiation [199, 257]. *LRP1* mRNA and protein are reported to be increased in neurons and GFAP-positive activated astrocytes associated with neuroinflammation in AD patients [258, 259]. Meanwhile, a decrease of LRP1 has also been reported in the midfrontal cortex of AD patients, playing a role in modulating Aβ deposition and AD susceptibility [260]. In addition, in the APP23 mouse model, LRP1 is increased in the cortex but decreased in the vascular endothelial cells, which may account for the imbalance between Aβ efflux and influx across the BBB [261]. The level of BRI2 containing the BRICHOS domain is increased in the hippocampus of early-stage AD patients, whereas the level of the BRI2-APP complex is decreased, accompanied by a decrease of furin, indicating that the aberrant processing of BRI2 may promote its deposition and affect its function in halting Aβ production and aggregation [212]

#### Potential role of furin in AD pathology

**Fig. 3:**
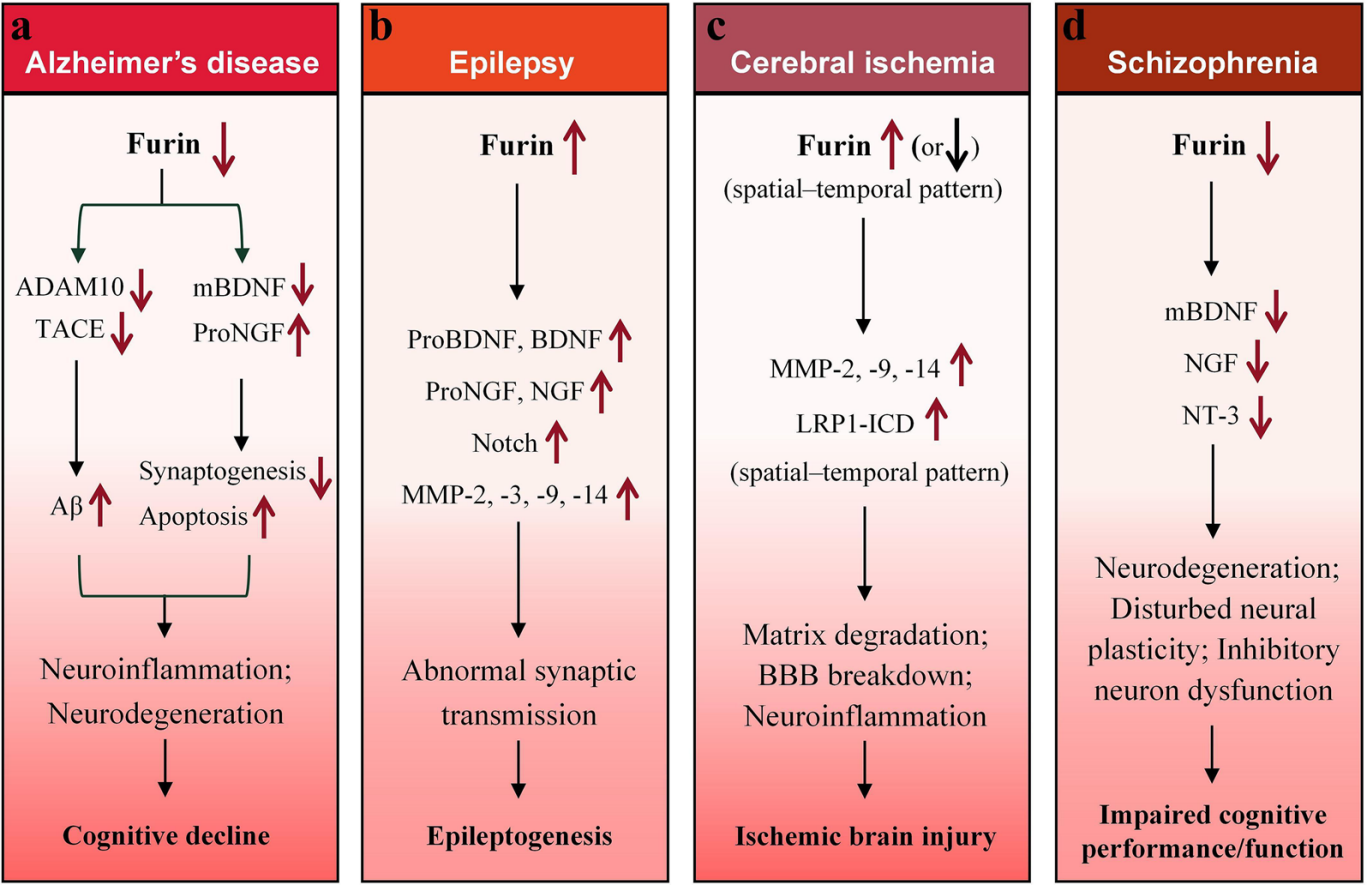
Proposed working models of how aberrant furin expression participates in the pathogenesis of Alzheimer’s disease (**a**), epilepsy (**b**), cerebral ischemia (**c**) and schizophrenia (**d**)

The above findings suggest an important role of furin in AD pathology. The downregulation of furin in AD patients or animal models likely leads to lower cleavage of ADAM10, TACE, proBDNF and proNGF. The decreased ADAM10 and TACE lead to reduced α-secretase activity, which in turn promotes Aβ generation and deposition; on the other hand, the low levels of mBDNF and high levels of proNGF cause neuronal death and synaptic damage (Fig. 3a). These alterations can account in part for the pathological symptoms of AD. In addition, the relationships between furin deregulation and changes in MMPs and LRP1 in AD pathology have yet to be investigated, and the causes of furin downregulation in AD need to be clarified.

### Furin in PD

#### PD overview

PD is the second most common neurodegenerative disorder, pathologically characterized by abnormal deposition of α-synuclein aggregates in Lewy bodies and loss of nigrostriatal DA neurons [262, 263]. The striking clinical symptoms of PD are motor symptoms such as tremor, rigidity, bradykinesia and postural instability. Patients with severe motor symptoms often have difficulties moving their hands, or standing and walking due to the tremor and stiff muscles, which severely affects the quality of their lives [263]. PD patients also display non-motor signs and symptoms, such as olfactory loss, autonomic dysfunction and rapid eye movement sleep behavior disorder, which usually precede the motor symptoms but are often overlooked [264]. The mechanism of neurodegeneration in PD remains unclear, and currently there is no cure for PD.

#### Aberrant furin expression in PD

Currently, there is no report on the changes of furin expression in PD patients or murine models. However, in Parkinson’s-related *Drosophila* model, furin 1 has been found to be highly concentrated in TH-positive DA neurons [265], and furin 1 is translationally regulated by leucine-rich repeat kinase 2 (LRRK2) and involved in the impairment of synaptic plasticity and neurodegeneration [266]. In addition, in the paraquat-induced *Drosophila* model of PD, furin 1 expression is also enhanced by paraquat exposure in DA neurons [265]. These results highlight a potential role of furin in PD pathogenesis.

#### Expression of substrates of furin in PD

Aberrant BDNF expression has been found during the pathological processes of PD. The amount of 27-kDa BDNF is increased in the CSF samples of PD patients compared with normal controls [267], whereas serum BDNF levels are significantly lower in PD patients than in healthy controls, which are correlated with motor impairment and cognitive deficits in PD [268, 269]. MMP-2 levels are reduced in PD patients in the substantia nigra, but not in the cortex and the hippocampus [131]. MMP-3 levels are increased in a rat model of PD induced by injection of 6-hydroxydopamine into the substantia nigra [129], and MMP-3 may play a pivotal role in the progression of PD through digestion of α-synuclein in DA neurons and modulation of α-synuclein aggregation and Lewy body formation [129, 130]. Serum MMP-1 is significantly lower in PD patients than in controls, and the difference is more prominent in females [270]. Both mRNA and protein of *GPR37* accumulate in Lewy bodies in the midbrain of PD patients [181, 182], and the increased Ecto-GPR37 in CSF is proposed as a potential biomarker for PD [182]. However, no studies currently exist regarding the specific relationship between furin expression and changes in these substrates in PD patients or animal models. Thus, deeper exploration of the underlying mechanisms remains essential in future studies, which may uncover new therapeutic targets for PD.

#### Potential role of furin in PD pathology

Although there is no report on the change of furin expression in vertebrate models of PD, the highly increased furin 1 expression in DA neurons of Parkinson’s-related *Drosophila* model indicates a potential role of furin in PD pathology. Furthermore, changes in the expression of some substrates of furin have been detected in PD models, such as increased GPR37 and MMP-3, highlighting the possible associations between furin and PD symptoms. Thus, the expression of furin in PD pathogenesis and associations with the change of its substrates in PD need to be clarified urgently.

### Furin in epilepsy

#### Epilepsy overview

Epilepsy is a common chronic neurological disorder associated with abnormal synaptic transmission [271], inappropriate neuronal firing, and imbalance of excitation and inhibition of neuronal networks [272]. The etiology of epilepsy is mostly unclear, which possibly includes genetic risks, brain diseases, and systemic diseases. The abnormal neuronal firing is found to be closely related to mitochondrial dysfunction and abnormalities in neurotransmitters and ion channels [273, 274]. Due to the different starting sites and transmission modes of abnormal neuronal discharges, clinical manifestations of epilepsy are complex and diverse, including disorders in motor, sensory, and autonomic nervous systems and consciousness [275].

#### Aberrant furin expression in epilepsy

It has been reported that furin protein levels are increased in the temporal neocortex of patients with temporal lobe epilepsy (TLE) and in the cortex and hippocampus of kainic acid (KA)-induced and pentylenetetrazol (PTZ)-kindled epileptic mice [25]. Moreover, transgenic overexpression of furin in mice increases the susceptibility to epilepsy and increases the epileptic activity [25]. Furin has been identified to play a role in influencing the inhibitory synaptic transmission in epileptic mice [25]. In addition, an increase in *Furin* mRNA has been found in the hippocampus of KA-exposed  mice [12], and the co-localizations of the increased *Furin* mRNA with *Ngf* and *Bdnf* mRNAs suggest a potentially important role of furin in the pathophysiology of epilepsy [12].

#### Expression of substrates of furin in epilepsy

Studies on animal models of epilepsy have proposed potential involvement of dysregulations of neurotrophins, such as BDNF, NGF and NT-3, in human epilepsy [276–282]. TLE patients with hippocampal sclerosis show increased mRNA levels of *BDNF*, *NGF* and *NT-3* in granule cells of hippocampus, which are correlated with either hippocampal neuron loss or aberrant supragranular mossy fiber sprouting [276]. Patients with intractable TLE show a marked increase in protein levels of BDNF in the temporal neocortex [277]. Moreover, a rapid increase in the proBDNF level is found in principal neurons and astrocytes of all hippocampal subfields in pilocarpine-induced status epileptic mice, which is proposed to be associated with the reduced proBDNF cleavage machinery [278]. Similar to the changes in BDNF, *Ngf* mRNA increases in the hippocampus and neocortex of rats with limbic seizures [279]. The secreted proNGF is considered as a pathophysiological death-inducing ligand [280], while blocking proNGF can inhibit neuronal loss after seizures [281]. Notch signaling is activated in KA-induced epileptic mice and in human epileptogenic tissues, while activation of Notch signaling further promotes neuronal excitation of CA1 pyramidal neurons [163]. In addition, a large number of studies have shown that the expression levels of MMP-2, MMP-3, MMP-9 and MMP-14 in the brains of epilepsy patients or animal models are increased and dynamically regulated at different stages of epileptogenesis [283–289]. MMP inhibitors are considered as potential therapeutic drugs because of their anti-seizure and anti-epileptogenic effects [285, 290].

#### Potential role of furin in the pathology of epilepsy

The above findings suggest a crucial role of furin in the pathology of epilepsy. The upregulation of furin in epilepsy patients or animal models may promote the cleavage of proBDNF, proNGF, Notch receptor and MMPs. As a result, the inhibitory and excitatory synaptic transmissions are affected, leading to abnormal neuronal discharge, which contributes in part to the symptoms of epilepsy (Fig. 3b). However, the underlying mechanisms for furin upregulation and furin-mediated activities in epileptogenesis need to be determined.

### Furin in cerebral ischemia

#### Overview of cerebral ischemia

Cerebral ischemia is a neurodegenerative disease caused by reduced blood supply to the brain tissue [291], and is currently a major cause of death and disability globally [292]. Cerebral ischemia causes reduced delivery of oxygen and glucose to the brain, and as a result, a loss of consciousness can occur [291]. The occurrence of metabolic disorders during ischemia or tissue hypoxia is relatively well established, but the subsequent reperfusion is the major events leading to cell and tissue dysfunctions [293]. Ischemia–reperfusion injury is the inexplicable aggravation of cellular dysfunction during the restoration of blood flow after a period of ischemia [294]. The reperfusion can lead to potentially very harmful effects, such as necrosis of irreversibly damaged cells, cell swelling, vascular and endothelial injury and mitochondrial dysfunction [295].

#### Aberrant furin expression in cerebral ischemia

It has been found that the *Furin* mRNA level in rat hippocampus at 24 h after transient global cerebral ischemia is two-fold of that in sham-operated controls, indicating a possible role furin may play [296]. In a focal ischemic rat model established by middle cerebral artery occlusion, increases in *Furin* mRNA and protein levels are found in the piriform cortex of the ischemic hemisphere 2 h after reperfusion compared with sham-operated animals, and it is predicted that the elevation of furin may contribute to the disruption of BBB during ischemia [297]. Another recent study found that the level of *Furin* mRNA in the ipsilateral cortex of hypoxic-ischemic rats had an insignificant increase at 6 h after ischemia, but then decreased significantly at 15 h and was sustained at a low level for 7 days [298], while *Furin* mRNA in the ipsilateral hippocampus was elevated at 6 h and 3 days but decreased at 15 and 24 h after injury compared with that of the control rats [298]. The change in furin expression is considered to account for the decrease of BDNF in the ipsilateral cortex and hippocampus of the rats [298]. An in vitro study also showed that the protein levels of furin and BDNF are upregulated in cultured rat astrocytes exposed to oxygen–glucose deprivation [64]. These findings indicate that furin may play important roles in the pathogenesis of cerebral ischemia and in the recovery from ischemia brain damage.

#### Expression of substrates of furin in cerebral ischemia

In addition to the changes in furin expression, the levels of Bdnf mRNA and protein in the ipsilateral cortex and hippocampus of hypoxic-ischemic rats are altered at different degrees at different time points after hypoxic-ischemic injury [298]. Many other studies have also reported changes of MMP levels, including levels of MMP-2, MMP-9 and MMP-14, in the model of focal ischemic rats [297, 299–302]. In particular, increased expression and activity of MMP-2 and MMP-9 are found in different models of focal cerebral ischemia, implying their potential roles in early matrix degradation, loss of vascular integrity, and neuronal injury in the ischemic lesion [300, 301]. In addition, a significant increase in the cleavage of LRP1 by furin has been found in rats after cerebral ischemia, which is predicted to aggravate neuroinflammation, and administration of a furin inhibitor inhibits the cleavage of LRP1 and decreases co-localization of ICD of LRP1 with furin in ischemic areas [303]. These findings imply that the furin-mediated cleavage of MMPs and LRP1 may be involved in the pathophysiology of ischemic brain injury.

#### Potential role of furin in the pathology of ischemia

The above observations imply the involvement of furin in the pathology of cerebral ischemia. Changes in furin expression may exist in varied temporal and spatial patterns after ischemic injury in the brain. The upregulation of furin in ischemic patients or animal models may promote the cleavage of MMPs, particularly MMP-2, MMP-9, and MMP-14. The activation of these MMPs leads to early matrix degradation and loss of vascular integrity, and finally contributes to BBB breakdown and neuronal injury in ischemic lesions (Fig. 3c). Moreover, the ICD of LRP1 is increased, which aggravates neuroinflammation. The relationship between changes of furin level and other molecules such as BDNF in ischemic brain injury needs to be elucidated in the future.

### Furin in SCZ

#### SCZ overview

As one of the severe mental diseases, schizophrenia is characterized by cognitive distortions including impairments in concentration, thinking, speed of cognitive information processing, and verbal working memory [304]. These impairments in cognitive functions persist throughout the disease and determine the functional status of patients [305]. The etiology of schizophrenia is complex, commonly associated with genetic variants and changes in development-related factors and regulatory molecules [306].

#### Aberrant furin expression in SCZ

A study by Fromer et al. in 2016 using RNA sequencing data from the dorsolateral prefrontal cortex of post-mortem SCZ patients identified down-regulation of *FURIN* transcripts by risk allele [24]. They also found that depletion of furin in zebrafish model has the largest impact on head size, which can be attributed to the furin depletion-induced changes in neural cell proliferation and migration [24]. Furthermore, downregulation of furin expression specifically at the rs4702 G (in the 3' UTR of *FURIN*) allele by miR-338-3p reduces the production of mBDNF [307]. In addition, the association between pleiotropic effects of *FURIN* genetic loci and SCZ traits has been reported recently by several different studies [308–310]. A study using datasets from the Psychiatric Genomics Consortium related to SCZ, major depressive disorder (MDD) and bipolar disorder (BIP) patients identified rs8039305 in the *FURIN* gene as a novel pleiotropic locus across the three disorders [309]. Similarly, another study identified rs17514846, a variant within an intron of *FURIN* gene, as a common trait between SCZ and cardiometabolic disorder [310]. In addition, in *C. elegans*, the 3'UTR of *kpc-1* (furin) promotes dendritic transport and local translation of mRNAs to regulate dendrite branching and self-avoidance [311]. These findings indicate the important role of furin in brain development and in the pathophysiology of SCZ.

#### Expression of substrates of furin in SCZ

The deregulation of BDNF expression has been extensively studied in SCZ patients and animal models [312–319]. Significant reductions of *BDNF* mRNA and protein have been observed in the dorsolateral prefrontal cortex of patients with SCZ compared to normal individuals [312]. The reduced BDNF/TrkB signaling in the prefrontal cortex appears to underlie the dysfunctions of inhibitory neurons in subjects with SCZ [313]. Studies have also shown significant reductions of BDNF in the hippocampus as well as NT-3 concentrations in the frontal and parietal cortical areas, in SCZ patients [314]. On the contrary, some studies have shown that the BDNF concentration is significantly increased in cortical areas of post-mortem SCZ patients [314, 315]. In addition, the plasma BDNF levels in schizophrenic patients are remarkably lower than those in the controls, which is predicted to be associated with the decreased hippocampal volume and cognitive impairments in first-episode and chronic SCZ [316, 317]. These findings suggest that the downregulation of neurotrophic factors could be responsible for neural maldevelopment and disturbed neural plasticity in the etiopathogenesis of schizophrenic psychoses. In schizophrenic animal models, reductions of *Bdnf* mRNA and protein levels have been observed in the cortex and the hippocampus [318, 319]. Decreased serum levels of NGF and NT-3 have been observed in SCZ as well [320–322]. In addition to the alterations of neurotrophins, plasma MMP-9 levels are also increased significantly in SCZ patients compared to controls [323], and MMP-9 gene polymorphisms in the brain are found to be associated with SCZ [324, 325]. Besides, increased MMP-2 levels in the CSF of SCZ patients are also reported [326].

#### Potential role of furin in SCZ pathology

The above findings uncover the involvement of furin in the pathology of SCZ. Furin expression in SCZ patients is downregulated, which in turn affects the maturation of neurotrophins, such as BDNF, NGF and NT3. The chronic low trophic support for neurons leads to neural maldevelopment, dysfunction of inhibitory neurons, disturbed neural plasticity and neurodegeneration, contributing to the impaired cognitive performance/function in SCZ (Fig. 3d). This hypothesis may in part explain the pathogenesis of SCZ. However, the relationships between furin deregulation and changes in MMPs and other furin substrates in SCZ pathology have yet to be investigated.

### Furin in depression and anxiety

Currently, there is no report on the changes of furin expression in patients with depression and anxiety. However, the SNP rs8039305 in the *FURIN* gene has been indicated as a novel pleiotropic locus across the disorders of MDD, BIP and SCZ [309], indicating a potential role of furin in pathological mechanisms of the psychiatric disorders.

Aberrant expression of several substrates of furin has been reported in patients with depression. The serum BDNF level is significantly lower in MMD patients than in healthy controls [327–329]. The mBDNF/proBDNF ratio is also decreased [329], suggesting that the reduced BDNF maturation plays a pivotal role in the pathophysiology of MDD. Serum MMP-9 is found to be increased in MDD patients, while MMP-2 is decreased in MDD patients [323, 330], indicating the involvement of MMP-2 and MMP-9 in mood disorders. In addition, MMP-2 levels in the CSF are increased in MDD patients [326], and the state-dependent alterations of MMP-2 and activation of cascades involving MMP-2, MMP-7, and MMP-10 appear to play a role in the pathophysiology of MDD [326]. LRP1 has been reported to be up-regulated in the hippocampus of depressive-like rat model [331].

In anxiety-like disorders, aberrant BDNF expression has also been reported. In the social deprivation stress-triggered anxiety- and depressive-like mice, BDNF levels are reduced in the brain [332]. In serotonin transporter knockout rats with depressive- and anxiety-like behavior, a decrease in mBDNF in the prefrontal cortex has been reported as well [333]. The alterations of proBDNF and mBDNF expression have been indicated in many other diseases with anxiety- and depressive-like behavior [334–337], highlighting the association between aberrant BDNF expression and anxiety and depression disorders.

## Furin-targeting strategies for neurological diseases

Currently, the use of furin-targeting strategies to diagnose or treat neurological disorders has not been reported in clinical studies. However, as described above, furin expression levels are altered in several neurodegenerative and neuropsychiatric diseases; for instance, serum furin level is decreased in AD mice. These highlight the great potential of furin to be a predictive diagnostic marker for neurological disorders in the future.

The potentials of furin-targeting strategies to treat neurological diseases have been suggested in several animal models (Table 2). In AD animal models, injection of *Furin*-adenovirus into the cortex of Tg2576 mice markedly increases the α-secretase activity of ADAM10 and TACE, which in turn reduces Aβ production [13]. *Furin*-transgenic mice with brain-specific overexpression of furin exhibit increased dendritic spine density and enhanced learning and memory, which are attributed to the increased mBDNF level caused by furin [26]. In aged APP-C105 mice, treadmill exercise attenuates AD-related symptoms, possibly by ameliorating iron dyshomeostasis and enhancing furin expression, thereby promoting α-secretase-directed processing of APP [23]. Gallic acid treatment in APP/PS1 mice has been shown to increase furin expression, which in turn promotes α-secretase activity and decreases Aβ production, partly reversing the learning and memory impairment in APP/PS1 mice [338]. In addition, cerebrolysin, a peptidergic mixture with neurotrophic-like properties, can improve the survival of neural stem cell grafts and alleviate Aβ deposition in the hippocampus of APP transgenic mice, and this protective effect also involves the activation of furin and increased BDNF expression [339]. On the other hand, knockdown of astrocytic Grin2a in rats reduces furin expression and in turn decreases the maturation and secretion of NGF, aggravating the Aβ-induced memory and cognitive deficits [238]. These findings suggest the potential of increasing furin expression as an effective approach for AD treatment, and open avenues for future targets and strategies for AD prevention and therapeutic interventions.

**Table 2:** Treatment effects of modulation of furin expression on neurological diseases

Disease	Model	Treatment	Targeted region	Furin expression	Effects	References
AD	Tg2576 mice	Furin adenovirus	Cortex	Cortex ↑	Reduces Aβ production by increasing α-secretase activity of ADAM10 and TACE	[13]
	*Furin*-Tg mice	Brain-specific transgenic overexpression of furin	Brain	Brain ↑	Elevates production of mBDNF, enhances dendritic spine density and promotes learning and memory	[26]
AD	APP-C105 mice	Treadmill exercise	Whole body	Cortex ↑	Increases furin expression, promoted APP cleavage by α-secretase, and attenuates AD-related symptoms	[23]
AD	APP/PS1 mice	Gallic acid	Whole body	Brain ↑	Increases furin expression, activates ADAM10, and reverses the loss of learning and memory	[338]
AD	APP transgenic mice	Cerebrolysin	Hippocampus	Hippocampus ↑	Increases furin and BDNF expression, improves survival of neural stem cell grafts and alleviates Aβ deposition	[339]
PD	Paraquat-treated *Drosophila*	Transgenic knockdown of *Fur1*	DA neurons	DA neurons ↓	Protects DA neurons against the toxic effect of paraquat	[265]
PD	*Drosophila* with LRRK2 overexpression	Disruption one allele of *Fur1*	Whole body	Whole body ↓	Reduces the retrograde synaptic enhancement induced by postsynaptic overexpression of LRRK2	[266]
PD	*Drosophila* with LRRK2 overexpression	Postsynaptic knockdown of *Fur1*	Postsynaptic muscles	Neuromuscular junction ↓	Reduces the retrograde synaptic enhancement induced by postsynaptic overexpression of LRRK2	[266]
Epilepsy	KA-induced epileptic mice; PTZ-kindled epileptic mice	Lentivirus containing sh-*Furin*	Hippocampus	Hippocampus ↓	Reduces the spontaneous rhythmic electrical activity of cerebral neurons and suppresses epileptic seizure activity and severity	[25]
Cerebral ischemia	Global ischemia rats	Monosialoganglioside; Flavanol epicatechin	Whole body	Hippocampus ↑	Increases the levels of furin and NGF	[340]

In paraquat-induced *Drosophila* model of PD, transgenic knockdown of *Fur1* in DA neurons provides significant protection against the loss of DA neurons [265]. In *Drosophila* models with LRRK2 overexpression, disruption of one allele of *Fur1* or postsynaptic knockdown of *Fur1* using transgenic RNA interference approach can attenuate the LRRK2-induced retrograde synaptic enhancement [266]. These findings suggest potential involvement of furin in PD pathophysiology and treatment. However, great efforts are urgently needed to explore the role and pharmaceutical potential of furin in PD patients or murine models.

In both KA-induced and PTZ-kindled epileptic mouse models, lentivirus-mediated knockdown of furin in the hippocampus decreases the spontaneous rhythmic electrical activity of cerebral neurons, and suppresses epileptic seizure activity and severity [25]. This protective role is proposed to be associated with the regulation of synaptic transmission by altering the transcription level of postsynaptic gamma-amino butyric acid A receptor [25].

In a global ischemia/reperfusion rat model, monosialoganglioside or flavanol epicatechin treatment both can improve spatial memory retention and acquisition in experimental ischemic rats [340], and these neurotherapeutic effects are found to be related to the increases in furin and NGF levels [340]. In addition, application of furin inhibitor can protect primary cortical neurons from cell death induced by activated NMDA receptors [341], which is possibly attributed to the decrease of furin-mediated cleavage of LRP1 [303]. These findings suggest that manipulating furin expression is potentially a good strategy for the treatment of ischemic brain injury.

In addition, some furin activators and inhibitors have been identified with drug potentials. The small molecules phorbol esters dPPA (12-deoxyphorbol 13-phenylacetate 20-acetate) and dPA (12-deoxyphorbol 13-acetate) exhibit great effects in promoting furin expression via activation of the transcription factor CEBPβ in neuronal cells [34]. On the other hand, polyarginines, such as hexa-*D*-arginine, significantly inhibit furin activity in vivo [342, 343]. The therapeutic effects of these furin activators and inhibitors in prevention and treatment of neurological disorders need to be investigated further in the future.

## Conclusions

A growing body of evidence has suggested the crucial role of furin in the pathophysiological conditions of neurodegenerative and neuropsychiatric diseases. Notably, reduced furin expression is closely associated with the pathogenesis of AD. Pharmaceutical targeting of furin expression has shown great promise for AD treatment. In addition to AD, alterations of furin expression also exist in patients or animal models of epilepsy, cerebral ischemia, or SCZ. Furthermore, changes in the expression of neurotrophins, such as BDNF and NGF, are common to these neurodegenerative and neuropsychiatric diseases, and many are related to the abnormal cleavage of proneurotrophins. In addition to neurotrophins, other substrates of furin such as MMPs and LRP1 also exhibit expression changes in these neurodegenerative and neuropsychiatric diseases. These lines of evidence highlight the important roles of furin and furin-mediated activities in the progression of these diseases, and render furin as a valuable therapeutic target. However, currently very little is known about the cellular and molecular mechanisms of furin regulation in these diseases. Future studies are needed to clarify the molecular mechanisms of furin deregulation and its involvement in the pathogenesis of these diseases, and to develop new diagnostic and treatment strategies.

## Data Availability

Available upon request.

## Abbreviations

AD: Alzheimer’s disease
SCZ: Schizophrenia
PC: Proprotein convertase
BDNF: Brain-derived neurotrophic factor
NGF: Nerve growth factor
MMPs: Multiple matrix metalloproteases
PD: Parkinson’s disease
HIF-1: Hypoxia-inducible factor-1
TGFβ1: Transforming growth factor beta1
ER: Endoplasmic reticulum
DG: Dentate gyrus
ADAMs: A disintegrin and metalloproteases
BACE1: Beta-site APP cleaving enzyme 1
LRP1: Low-density lipoprotein receptor-related protein 1
GPR37: G-protein-coupled receptor
mBDNF: Mature BDNF
TrkB: Tropomyosin-related receptor kinase B
PI3K: Phosphatidylinositol 3-kinase
Akt: Protein kinase B
PLCγ: Phospholipase C-gamma
CaMK: Calcium-dependent protein kinase
MAPK: Mitogen-activated protein kinase
LTP: Long-term potentiation
p75NTR: P75 neurotrophic receptor
mNGF: Mature NGF
TrkA: Tropomyosin-related receptor kinase A
NT-3: Neurotrophin-3
NT-4/5: Neurotrophin-4/5
DA: Dopaminergic
APP: Amyloid-β precursor protein
sAPPα: αAPP
Aβ: Amyloid-β
CSF: Cerebrospinal fluid
ICD: Intracellular domain
NMDA: N-methyl-D-aspartate
BRI2: Integral membrane protein 2
NFTs: Neurofibrillary tangles
BBB: Blood–brain barrier
TACE: Tumor necrosis factor-α converting enzyme
LRRK2: Leucine-rich repeat kinase 2
TLE: Temporal lobe epilepsy
KA: Kainic acid
PTZ: Pentylenetetrazol
MDD: Major depressive disorder
BIP: Bipolar disorder
